# Is increased fat content of hindmilk due to the size or the number of milk fat globules?

**DOI:** 10.1186/1746-4358-4-7

**Published:** 2009-07-16

**Authors:** Katsumi Mizuno, Yoshiko Nishida, Motohiro Taki, Masahiko Murase, Yoshiharu Mukai, Kazuo Itabashi, Kazuhiro Debari, Ai Iiyama

**Affiliations:** 1 Department of Pediatrics, Showa University of Medicine, 1-5-8 Hatanodai, Shinagawa-ku, Tokyo, Japan 142-8666; 2 Department of Dental Hygiene, Showa University of Medicine, Tokyo, Japan; 3 Department of EM-Laboratory, Showa University, Tokyo, Japan; 4 Nikkiso Co. Ltd. Tokyo, Japan

## Abstract

**Background:**

It is known that the fat content of breast milk is higher in hindmilk than in foremilk. However, it has not been determined if this increased fat content results from an increase in the number of milk fat globules (MFGs), an increase in the size of MFGs, or both. This study aims to determine which factor plays the most important role.

**Methods:**

Thirteen breastfeeding mothers were enrolled in the study and we obtained 52 samples from 26 breasts before (foremilk) and after (hindmilk) a breastfeeding session. The fat content was evaluated by creamatocrit (CrCt) values. MFG size was measured with the laser light scattering method. We compared CrCt values and MFG size between foremilk and hindmilk.

**Results:**

Although the CrCt values were higher in the hindmilk (8.6 ± 3.6%) than in the foremilk (3.7 ± 1.7%), the MFG size did not change (4.2 ± 1.0 μm and 4.6 ± 2.1 μm, foremilk and hindmilk, respectively). There was no relationship between the changes in CrCt versus MFG size from foremilk to hindmilk.

**Conclusion:**

The results indicate that the increase in fat content results mainly from the increased number of MFGs, which may be released into the milk flow as the mammary lobe becomes progressively emptied.

## Background

Fat is one of the most important nutrients for neonates as it comprises approximately 55% of the total energy of milk. It is well known that the fat content increases from the beginning to the end of a feed, resulting in an increase in the total calories of hindmilk. Daly et al. determined that most of the variation in milk fat content within women results from the degree of breast fullness [1]. Several theories have speculated on the reasons why fat content increases as the breast gets progressively emptied. Whittlestone proposed a fat globule filtration effect whereby fat globules cluster in the lumen of the alveolus and are removed only towards the end of the feed [2]. Hytten suggested that the increase is a result of fat globules adsorbing the large alveolar and duct surface of the full alveolus, only being removed towards the end of the feed [3]. From their study on sows, Atwood and Hartmann postulated that the removal of adsorbed fat globules is the result of the changing morphology of the lactocyte and the shape of the alveolus [4]. When the lumen is full, lactocytes are squamous in shape, providing maximal surface area for the fat globules to adsorb. As the lumen drains, the lactocytes change from squamous to columnar. This subsequent decrease in luminal surface area together with the mechanical shearing forces generated by the convolution of the alveolus displaces the adsorbed fat globules, facilitating their removal. This theory also explains the previously noted rapid rise in fat content as more milk is removed from the gland [1]. This issue remains unclear in humans however.

More than 98% of the fat in breast milk is composed of triacylglycerols [5-7]. The nonpolar nature of milk lipids prevents solubility in the aqueous phase, within the mammary secretory cells before secretion, as well as in milk [8]. Milk lipids originate as small droplets of triacylglycerol, synthesized in or on the surfaces of rough endoplasmic reticulum membranes. These droplets are released into the cytoplasm as microlipid droplets with a surface coat of protein and polar lipid. The microlipid droplets fuse with each other to form larger cytoplasmic lipid droplets. Droplets of varying size are transported to the apical cell membrane and are extruded into the alveolar lumen [9-14]. During the extrusion process, the globule is enveloped by portions of the cell membrane, which becomes the milk fat globule (MFG) membrane.

There are three possible mechanisms involved in increasing the fat content from foremilk to hindmilk. Firstly, the number of MFGs may increase toward the end of a breastfeeding session; secondly, lactocytes may produce larger MFGs as the lumen gets emptied; and thirdly, both factors may contribute. Although the first mechanism has been considered most plausible, this theory has not yet been proven. We conducted this study to determine which mechanism plays the most important role in increasing the fat content. In this study, we measured the MFG size distribution in foremilk and hindmilk in comparison with creamatocrit (CrCt) of the foremilk and hindmilk.

## Methods

### Sample size considerations

We assumed that the mean MFG size is 4 ± 1 μm and determined the statistical power as 0.9. Although it is difficult to estimate the change in CrCt during the experimental feed, in our breastfeeding center we found that hindmilk has twofold higher CrCt than foremilk [15]. If we assume that the 50% increase in CrCt results from the change in the size of MFGs, the MFG size is expected to be 6 μm. Therefore, we assumed that a more than 2 μm difference in MFG diameter between foremilk and hindmilk would be significantly different. Thus, to calculate the power, 14 samples were necessary. We collected an additional 12 samples from six women to ensure an adequate sample size.

### Subjects

This study was conducted between June 2007 and October 2007. Thirteen exclusively or nearly exclusively breastfeeding mothers were enrolled in the study at between one and two months postpartum. We obtained samples from 26 breasts. The subjects had no major breastfeeding problems or health issues. The characteristics of the mothers are listed in Table 1. Their infants were growing well on breast milk. This assessment was performed at the Breastfeeding Research Center in Showa University Hospital. When mothers visit the center, we discuss the important aspects of breastfeeding and the activities of the Breastfeeding Research Center. If mothers without problems expressed interest in our research, we explained the details of the research project to them and they then went on to make an appointment for an assessment. Mothers with significant breastfeeding-related problems were not enrolled in the study. Mothers were asked not to breastfeed their infants for one hour prior to the visit. The protocol was approved by the institutional review board of Showa University of Medicine and participants signed informed consent forms.

**Table 1:** Characteristics of participants (n = 13)

	**Mean ± SD**
Maternal age (year)	29.2 ± 3.1
Time postpartum (infant age) (weeks)	5.6 ± 0.7
Number of children including this infant	one: 10
	two: 3
Total mother breastfeeding duration (month)	3.2 ± 5.2
Present breastfeeding duration (weeks)	5.6 ± 0.7
Exclusive breastfeeding duration (weeks)	4.7 ± 1.2
Lactation frequency (feeds/24 hours)	9.9 ± 1.5
Number of feeds during day time	7.2 ± 0.9
Number of feeds during night time (10 pm-6 am)	2.8 ± 0.8
Usual breastfeeding duration (minutes)	27 ± 5
Volume ingested during the experiment (mL)	85 ± 45

### Sample collection and storage

All samples were collected under the supervision of International Board Certified Lactation Consultants by manual expression in microtubes (capillary tubes: 75 mm in length, plain, Drummond Co., Ltd. Tokyo, Japan) without preservative. Samples were collected before breastfeeding (foremilk) and after breastfeeding (hindmilk); a breastfeeding session was considered complete when the infant stopped sucking. The samples were stored at 4°C until they were analyzed in the laboratory the day after collection. We measured the MFG size distribution in foremilk and hindmilk in comparison with creamatocrit (CrCt) of the foremilk and hindmilk.

### Creamatocrit measurement

For the measurement of CrCt values, we centrifuged the milk samples in micro-hematocrit tubes at 12,000 g for 5 minutes to separate the cream layer (fat) from the skim milk. The cream layer was measured with a Vernier caliper and the percentage of total volume was calculated [16].

### Measurement of milk intake

Milk intake during breastfeeding was calculated using accurate test-weighting procedures with an electronic infant scale (Tanita Co. Ltd, Tokyo, JAPAN.). We weighed clothed infants pre- and post-feed under identical conditions and then subtracted the pre-feed from the post-feed weight [17].

### Measurement of MFG size

The measurement of MFG size by laser light scattering has been described elsewhere [18]. Microtrac^® ^S3500 (Nikkiso, Tokyo, Japan) was used for the laser light scattering method. It has particle size measurement capabilities ranging from 0.02 to 2800 μm. Microtrac S3500 utilizes Tri-Laser Technology for particle size measurement. The Tri-Laser System allows light scattering measurements to be made from the forward low angle region to almost the entire angular spectrum (approximately zero to 160 degrees). It achieves this through a combination of three lasers and two detector arrays, all in fixed positions. The analysis of scattered light to determine particle size employs a Mie-based unified angular scattering theory from large particle analysis to small particle analysis.

Standard parameters were calculated by the software: the mode diameter (diameter at the peak maximum); the volumic average diameter [Σ(vi × di)/Σvi (where vi is the volume of globules in a size class of average diameter di]; and the volume-surface average diameter (Σvi/Σvi/di): the specific surface area [SSA = 6 × ρ-1 × (Σvi/Σvi/di)-1, where ρ is the milk fat density (0.92 at 20°C)].

### Statistical analysis

Values are expressed as mean ± SD. In terms of statistical analysis, we used STATCELL 2007 (Fusou Co. Ltd., Tokyo, Japan). The paired t-test was applied for statistical analysis. p < 0.05 was considered significant.

## Results

The demographic characteristics of participants are listed in Table 1. For 10 mothers, this was their first infant. Nine mothers breastfed their infants exclusively and four mothers offered formula once or twice a day. Infant age was 5.6 ± 0.7 weeks on average. The milk volume ingested by infants from each breast ranged from 5 mL to 130 mL. As total intake, infants obtained 85 ± 45 mL (range 25 to 200 mL) of breast milk during the experiment.

**Figure 1:**
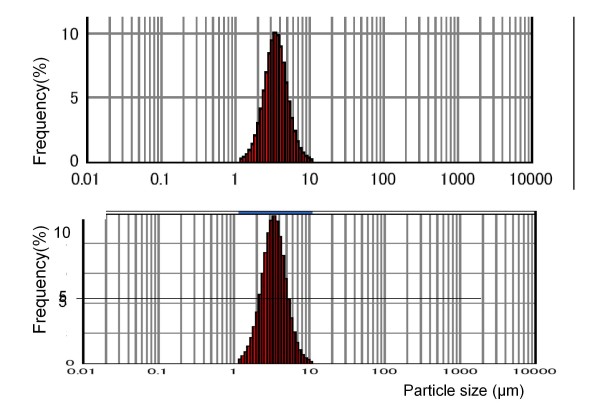
**Distribution of milk fat globule (MFG) particle sizes in foremilk and hindmilk**. The distribution of MFG sizes in milk from one mother is shown. The upper panel shows the distribution of MFG sizes in foremilk and the lower panel shows the distribution in hindmilk. The median particle size of this mother's milk is 3.4 *μ*m and 3.5 *μ*m, foremilk and hindmilk, respectively.

**Figure 2:**
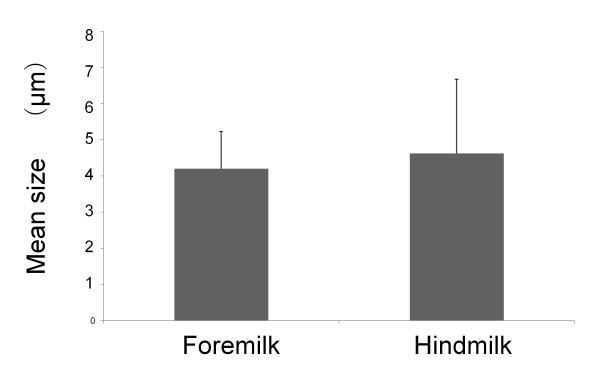
**Mean size of milk fat globule (MFG) foremilk vs. hindmilk**. The mean MFG sizes of foremilk and hindmilk. The Y-axis indicates the mean MFG size. There is no significant difference in the MFG sizes between foremilk and hindmilk.

**Figure 3:**
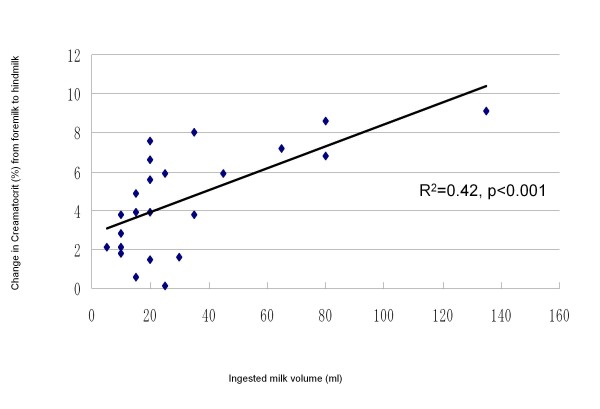
**Ingested milk volume and change in creamatocrit (CrCt) values**. The relationship between the ingested milk volume and change in CrCt values from foremilk to hindmilk is shown. The X-axis indicates the milk volume ingested by infants (mL). The Y-axis indicates the CrCt differences between foremilk and hindmilk. There is a significant relationship between two variables (r^2 ^= 0.42, p < 0.001).

The distribution of MFG sizes in milk from one mother is shown in Figure 1. Table 2 shows no significant difference in fat globule size parameters between foremilk and hindmilk. The mean values for the MFG diameter were 4.2 ± 1.0 μm and 4.6 ± 2.1 μm for foremilk and hindmilk, respectively (Figure 2). The CrCt values were 3.7 ± 1.7% and 8.6 ± 3.6% for foremilk and hindmilk, respectively. There was a significant difference in CrCt values between foremilk and hindmilk (p < 0.001), but the MFG size did not differ significantly between foremilk and hindmilk. There was no significant relationship between MFG size and CrCt. Although ingested milk volume did not show any relationship with MFG size, there was a significant positive relationship between the ingested milk volume and the change in CrCt values from foremilk to hindmilk (Figure 3, r^2 ^= 0.42, p < 0.001).

**Table 2:** Comparison between mode diameter (MD), volume average diameter (VAD), and specific surface area (SSA) of the fat globule population measured from foremilk and hindmilk (n 26)

	MD (μm)*	VAD (μm)*	SSA (m^2^/g)*
Foremilk	4.2 ± 1.0	4.5 ± 1.1	1.9 ± 0.3
Hindmilk	4.6 ± 2.1	4.9 ± 1.0	1.8 ± 0.2

*Mean ± SD

There were no significant differences between foremilk and hindmilk in any of the three parameters.

## Discussion

It is well known that fat content increases from the beginning of a feed to the end of a feed. We have confirmed that CrCt measurements correlate with total milk fat extraction by the Folch procedure [19]. In this study, there was a significant relationship between ingested milk volume and changes in CrCt values from foremilk to hindmilk, as expected. However, the ingested volume had only a 40% effect on the change in CrCt, probably because the storage capacity of the breast plays an important part in determining the CrCt value. Because the storage capacity of a breast differs significantly not only among individuals, but also between the right or left breast of the same woman [1], the ingested volume played some role in the change in milk fat content. The MFG size was not affected by either the ingested volume or the change in milk fat content. From the findings of the present study, it is evident that the increase in milk fat content during a feed is derived from the increase in the number of MFGs.

The MFGs in milk have been extensively investigated not only in dairy cows, but also in human milk. Rüegg and Blanc used a particle counter and found the presence of subpopulations of differently sized particles in breast milk [20]. Since then, the change in MFG size during lactation has been reported elsewhere [18,21,22], although the laser light scattering method has only recently been utilized. The MFG size changes over the course of lactation; it is larger in early colostrum than in transitional or mature human milk [18,21]. After two weeks postpartum, the number of the largest MFGs (8 to 13 μm) decreases and stays at a low level [21]. Michalski et al. demonstrated that the size becomes larger between 3 and 20 months, however, so we collected breast milk from mothers during the restricted period of between one and two months postpartum [18].

In terms of the volumic average diameter and the specific surface area of MFG, Michalski et al. reported that these values in mature human milk were 4.4 ± 0.2 μm and 1.9 ± 0.1 m^2^/g, respectively [18]. Rüegg and Blanc reported that they were 5.2 ± 0.4 μm and 1.4 ± 0.1 m^2^/g, respectively [20]. Although the duration of lactation at the time of study and methods for MFG size measurement are somewhat different from the present study, these values correspond to the previously reported data. Again, these measurements did not differ between foremilk and hindmilk such that the size of the MFG has not altered from the beginning to the end of a breastfeed.

Other factors that change the MFG size are hormones, such as prolactin and oxytocin [11], and frequency of milking. Wiking et al reported that the MFGs become larger in cows that are milked more frequently [23]. In terms of the relationship between milk fat content and MFG size in a single female, the MFG size increases with the increase in milk fat content. In this study, all milk samples were measured in the same way as that in the bovine study. Variations in MFG diameter among milk extracted in the morning, midday, and evening were not significant [18]. In addition, no significant difference in MFG size was found between the milk at the end of the feed and the milk expressed before the feed in three women. Whittlestone and Perrin also found no difference in MFG size at the beginning and end of feeding by microscopic observations [24]. These articles contained no further discussion related to this issue. From these findings, we speculate that, over a longer time span, the increase in fat content results from the increase in the MFG size, but during a feed, the increase in the fat content does not result from the increase in the MFG size. This study is the first to explore the relationship between milk fat content and MFG size during a single feed.

Recently, differently sized MFGs have been found to have different function by the microfiltration technique. The fatty acid composition of the core and the membrane of differently sized MFGs separated by microfiltration have been extensively examined not only in dairy cow milk [25-29], but also in human milk [30]. Michalski et al. recently found that, in comparison with human milk, the fat droplet structure in infant formula results in lower protection ability against PUFA oxidation and higher concentration of 4-HHE and 4-HNE [31]. This is due to the wider fat surface area of fat droplets in infant formula. In addition, the larger fat interface enhances lipolysis and lipid oxidation [32]. Nevertheless, the origin and secretion of milk lipids as well as the function of different sized MFGs remain unclear. The properties and/or function of different sized MFGs should be of interest in future study.

## Conclusion

The aim of this study was to determine whether the increase in fat content from foremilk to hindmilk was due to an increase in the number or size of MFGs. The increase in fat content from foremilk to hindmilk results from the removal of adsorbed fat globules rather than the change in the size of MFGs. This finding supports the theory of removal of adsorbed fat globules described by Atwood and Hartmann [4].

## Competing interests

The authors declare that they have no competing interests.

## Authors' contributions

Mizuno K. had primary responsibility for protocol development, mother screening, enrolment, and writing the manuscript. Take M, Murase M, Nishida Y has participated in the measurement of particle size of milk fat globule and analysis

Itabashi K, Debari K, Mukai Y, and Iiyama A has participated in the interpretation of data and drafting the manuscript. All authors read and approved the final manuscript.
